# Droplet attraction and coalescence mechanism on textured oil-impregnated surfaces

**DOI:** 10.1038/s41467-023-40279-w

**Published:** 2023-08-18

**Authors:** Haobo Xu, Yimin Zhou, Dan Daniel, Joshua Herzog, Xiaoguang Wang, Volker Sick, Solomon Adera

**Affiliations:** 1 Department of Mechanical Engineering, University of Michigan, Ann Arbor, MI USA; 2 Division of Physical Sciences and Engineering, King Abdullah University of Science and Technology (KAUST), Thuwal, Saudi Arabia; 3 Department of Chemical and Biomolecular Engineering, The Ohio State University, Columbus, OH USA; 4 Sustainability Institute, The Ohio State University, Columbus, OH USA

**Keywords:** Mechanical engineering, Fluid dynamics

## Abstract

Droplets residing on textured oil-impregnated surfaces form a wetting ridge due to the imbalance of interfacial forces at the contact line, leading to a wealth of phenomena not seen on traditional lotus-leaf-inspired non-wetting surfaces. Here, we show that the wetting ridge leads to long-range attraction between millimeter-sized droplets, which coalesce in three distinct stages: droplet attraction, lubricant draining, and droplet merging. Our experiments and model show that the magnitude of the velocity and acceleration at which droplets approach each other horizontally is the same as the vertical oil rise velocity and acceleration in the wetting ridge. Moreover, the droplet coalescence mechanism can be modeled using the classical mass-spring system. The insights gained from this work will inform future fundamental studies on remote droplet interaction on textured oil-impregnated surfaces for optimizing water harvesting and condensation heat transfer.

## Abstract (web summary)

Purposely patterned surfaces impregnated with oil harbor a range of unusual wetting phenomena. Experiments now reveal long-range attraction between millimeter-sized water droplets on such surfaces, leading to three-stage coalescence.

## Introduction

In nature, the ability to repel water is often a matter of life and death. This is true for insects (such as water striders) and hummingbirds which must avoid getting wet by water^1–4^. Similarly, the tendency of water and other fluids, such as blood and oil, to stick to surfaces poses serious problems in various industrial applications ranging from food processing^5^ to biofouling prevention^6^. Hence, there is a dire need to develop robust liquid-repellent surfaces for numerous engineering applications.

Nature’s solution for the liquid “stickiness” problem is to decorate the surface with micro/nanostructures (that is, lotus-effect superhydrophobic surfaces^7–10^), which can trap air to allow droplets to reside on a solid-gas composite surface (Cassie state)^11,12^. Even though significantly reduced compared to flat surfaces, micro/nanostructuring alone does not fully eliminate contact line pinning^13,14^; there is still pinning at the solid-liquid contact points at the top of the structures. Moreover, the trapped air within the pores becomes unstable particularly for low surface tension fluids, resulting in highly pinned sticky droplets (Wenzel state)^15^. Superhydrophobic surfaces also fail in underwater applications^16^ since the trapped air can diffuse into the surrounding fluid.

The shortcomings of the lotus-leaf-inspired superhydrophobic surfaces can be remedied by using micro/nanotextured oil-impregnated surfaces. By replacing the air in conventional superhydrophobic surfaces with a more viscous lubricant oil, a droplet immiscible with the underlying lubricant layer can slide off easily with a <1-2° tilt angle. This relatively new concept was first reported in a 2005 review paper^17^ which described a composite “hemi-solid, hemi-liquid” slippery surface with “non-measurable” contact angle hysteresis^18,19^. An analogy to the slippery nature of *Nepenthes* pitcher plant^20–22^ was later made in 2011, and the concept was further pursued independently by different research groups with the resulting surface commonly known in the literature as slippery liquid-infused porous surface (SLIPS) or lubricant-impregnated surface (LIS)^23,24^. When designed well, textured oil-impregnated surfaces not only repel a wide variety of liquids such as water and low surface tension fluids, but they can also recover from mechanical damage and fabrication defects by redistributing the lubricant and self-healing via capillary wicking^25–27^.

Since 2011, there has been a considerable body of research on the fluid dynamics of droplets on textured oil-impregnated surfaces^28–30^, including the role of wetting ridge on lubricant longevity, oil depletion rate, and droplet interactions^29,31,32^. A droplet residing on an oil-impregnated surface siphons oil and forms a wetting ridge around its base due to the imbalance of interfacial forces at the contact line^33^, leading to a wealth of phenomena not observed in other conventional non-wetting surfaces such as superhydrophobic surfaces. For example, droplets residing on lubricant infused surfaces can be actuated by manipulating the position and shape of the wetting ridge^34,35^. The presence of a wetting ridge enables surface tension mediated remote interaction between neighboring droplets^36,37^ akin to the cheerios effect^38,39^. There is, however, insufficient understanding of the governing physics of droplet-droplet interaction and coalescence mechanism on oil-impregnated surfaces, which motivated the current work.

In this study, using a combination of geometry-based analytical modeling and experiments, we give a full account of the coalescence process between two droplets placed few millimeters apart on textured oil-impregnated surfaces. The understanding gained from this work is highly relevant for important industrial processes such as water harvesting^40^ and condensation heat transfer^41^. For example, the droplet coalescence described here is an essential method by which droplets grow in size during condensation. Larger droplets can then be removed by gravity by overcoming surface tension forces. This process clears the surface and allows re-nucleation, growth, and departure of condensate droplets that will lead to substantial improvements in the heat transfer coefficient^42,43^.

## Results

**Fig. 1:**
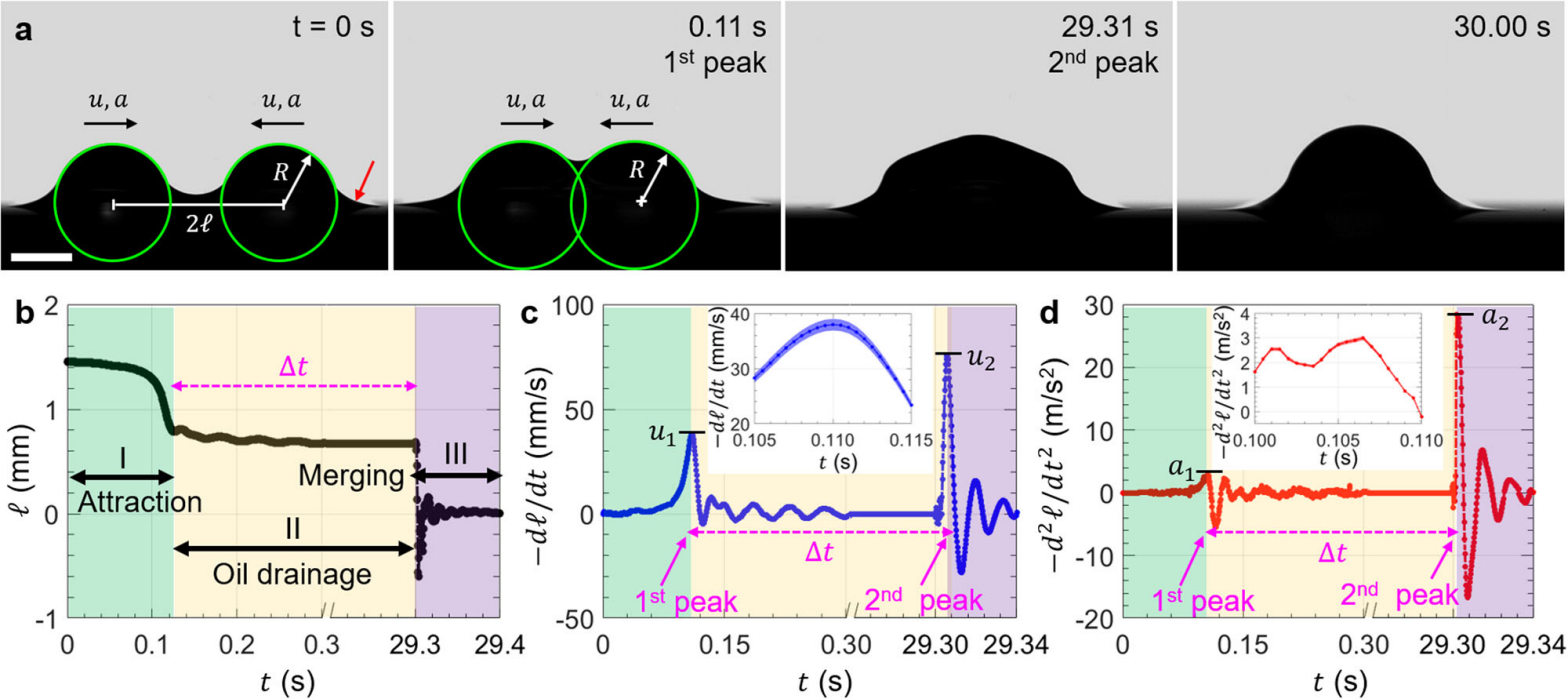
Droplet coalescence process. **a** Time-lapse images (scale bar = 1 mm) of surface tension mediated droplet attraction followed by coalescence captured at 5000 fps. Plots of instantaneous droplet position (**b**), velocity (**c**), and acceleration (**d**) as a function of time. Droplet coalescence occurs in three distinct stages: attraction when the wetting ridges meet/overlap (stage I), draining of oil between the droplets (stage II), and coalescence/merging (stage III). Droplet attraction (I) and coalescence (III) appear as peaks in velocity (${u}_{1}, {u}_{2}$) and acceleration (${a}_{1}, {a}_{2}$), whereas the time required to drain the oil in the wetting ridge (II) appears as a time-lag $\triangle t$ between the velocity and acceleration peaks. The shade in inset **c** and **d** shows 95% confidence interval of the droplet velocity and acceleration.

We start by placing two equally sized water droplets of radius $R$ ≈ 1 mm at a distance 2${{{{{\mathcal{l}}}}}}$ apart (Fig. 1a, scale bar = 1 mm) on a textured oil-impregnated surface (silanized silicon micropillars impregnated with silicone oil with dynamic viscosity ${\eta }_{o}$ = 10 cP; see scanning electron microscope images in Supplementary Fig. 1). An axisymmetric annular wetting ridge formed around the droplet base (red arrow, Fig. 1a) due to the imbalance of forces at the contact line^44,45^. Images of droplet interaction and coalescence were captured at 5000 frames-per-second (fps) using a high-speed camera (Phantom 1610, Vision Research). The distance 2${{{{{\mathcal{l}}}}}}$ between the center of mass of the two droplets was tracked as a function of time (Fig. 1b) by analyzing the time-lapse images and fitting the droplet outline with the circular Hough transform in MATLAB (green circles in Fig. 1a). Since the lubricant used in our experiments is relatively non-viscous (${\eta}_{o}$ = 10 cP) compared to water (dynamic viscosity ${\eta}_{w}$ = 1 cP), the wetting ridge can grow to its equilibrium size, i.e., the capillary length *l*_*o*_ = (γ_*o*_/*ρ*_*o*_*g*)^1/2^ ≈ 1.4 mm^18,45,46^, where γ_*o*_ = 19 mN/m is the surface tension of the lubricant oil measured using the pendant drop method, *ρ*_*o*_ = 930 kg/m^3^ is the density of oil, and *g* is the gravitational acceleration (*g* = 9.81 m/s^2^). The initial value of ${2{{{{{\mathcal{l}}}}}}}_{{{{{{\rm{init}}}}}}}$ ≈ 2.8 mm was chosen such that the wetting ridges of the two droplets overlap to initiate droplet interaction. The instantaneous velocity $u$ and acceleration $a$ of the droplet shown in Fig. 1c, d were obtained by taking the first and second derivative of the droplet position with respect to time using the finite difference method, i.e., $u={-{d}}{{{{{\mathcal{l}}}}}}/{dt}$ and $a={-{d}}^{2}{{{{{\mathcal{l}}}}}}/d{t}^{2}$. Unlike droplets on conventional textured superhydrophobic surfaces, droplets on textured oil-impregnated surfaces coalesce in three distinct stages as shown in Fig. 1b. The three stages of coalscence are denoted as stage I (attraction between the droplets), stage II (drainage of the oil in the wetting ridge), and stage III (coalescence or merging). The first stage (attraction) and last stage (coalescence) appear as peaks (local maxima) in velocity (${u}_{1}$ and ${u}_{2}$, Fig. 1c) and acceleration (${a}_{1}$ and ${a}_{2}$, Fig. 1d), while the second stage appears as a time-lag ($\triangle t$) between the velocity and acceleration peaks (Fig. 1b–d). This time-lag is also the time required to drain the oil in the wetting ridge before the two droplets coalesce. Using experiments that involve high-speed image analysis and theoretical modeling, this work focuses on advancing our understanding of the physical mechanisms and processes that govern the magnitudes of ${u}_{1}$, ${u}_{2}, {a}_{1}, {a}_{2}$, and $\triangle t$.

### Droplet attraction: first peak ${{{{{{\boldsymbol{u}}}}}}}_{{{{{{\boldsymbol{1}}}}}}}$ and ${{{{{{\boldsymbol{a}}}}}}}_{{{{{{\boldsymbol{1}}}}}}}$ (stage I)

**Fig. 2:**
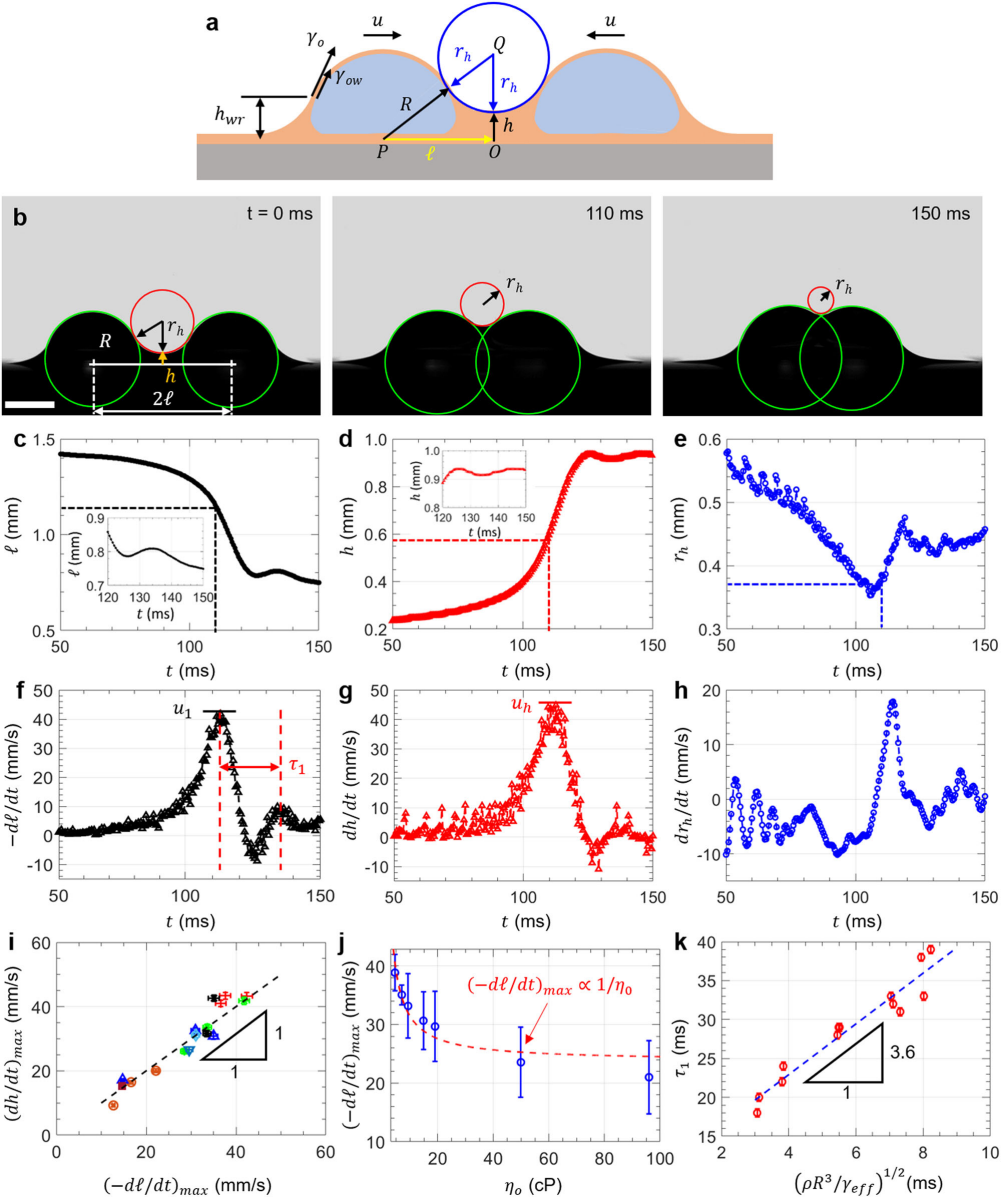
Surface tension mediated droplet attraction. **a** Schematic of two neighboring droplets forming a right triangle *OPQ*. **b** Time-lapse images of interacting droplets (scale bar = 1 mm), which are approximated as hemispheres with radius $R$. The oil meniscus separating the droplets is curve-fitted using a circular arc with radius ${r}_{h}$. The position of the droplet (**c**), the height of the oil meniscus (**d**), and the radius of the oil meniscus separating the droplets (**e**) are tracked using MATLAB by first converting them into binary followed by curve fitting using the Hough transform. Droplet approach velocity (horizontal) (**f**) and oil rise velocity (vertical) (**g**) as a function of time. The droplet approach peak velocity (${u}_{1}$) and oil rise peak velocity (${u}_{h}$) are approximately equal in magnitude. **h** The rate of change of the radius of the oil meniscus (${dr}_{h}/{dt}$) as the droplets approach each other reaches a maximum and decreases to zero near the first peak. **i** The peak approach velocity and oil rise velocity are approximately equal in magnitude irrespective of droplet size, oil viscosity, micropillar dimensions, and lubricant film thickness, which are represented with different data symbols in the figure. **j** The droplet approach velocity decreases as the lubricant oil becomes more viscous (${u}_{1} \propto 1/{\eta}_{o}$). **k** The period of oscillation (${\tau}_{1}$) scales with $({\rho} {R}^{3}/{\gamma}_{eff})^{1/2}$, a result that is analogous to the standard mass-spring harmonic oscillator. Error bars represent one standard deviation from repeated experiments.

Droplets interact with each other remotely and move towards each other when their wetting ridges meet. We first looked at the geometry of two droplets approaching each other (Fig. 2a). The droplets can be approximated as hemispheres (see Supplementary Fig. 2) with center *P* and radius $R$, while the oil meniscus separating the droplets was approximated using a circular arc with center *Q* and radius ${r}_{h}$. Connecting *P*, *Q*, and *O* (midpoint between the centers of droplet bases) gives a right triangle with legs *PO* (length ${{{{{\mathcal{l}}}}}}$) and *OQ* ($h+{r}_{h}$, where $h$ is the height of the oil meniscus), and a hypotenuse *PQ* ($R+{r}_{h}$). The three sides are related through the Pythagoras Theorem ${{{{{{\mathscr{l}}}}}}}^{2}+{\left({r}_{h}+h\right)}^{2}={\left(R+{r}_{h}\right)}^{2}$. When the droplets approach each other (Fig. 2b), ${{{{{\mathcal{l}}}}}}$ decreases (Fig. 2c), the meniscus height ($h$) increases (Fig. 2d), and the meniscus radius ${r}_{h}$ correspondingly decreases (Fig. 2e) before leveling off as the droplets collide at time $t$ ≥ 110 ms. Our experiments show that triangle *OPQ* remains a right triangle until the first peak at $t$ ≈ 110 ms. Taking the time-derivative of the Pythagoras relation gives ${{{{{\mathscr{-}}}}}}{{{{{\mathscr{l}}}}}}\frac{d{{{{{\mathscr{l}}}}}}}{{dt}}=\left(h-R\right)\frac{d{r}_{h}}{{dt}}+\left(h+{r}_{h}\right)\frac{{dh}}{{dt}}$, where $-d{{{{{\mathscr{l}}}}}}{{{{{\mathscr{/}}}}}}{dt}$ is the horizontal velocity at which the droplets approach each other (Fig. 2f), ${dh}/{dt}$ is the vertical velocity of oil rise in the wetting ridge (Fig. 2g), and $d{r}_{h}/{dt}$ is the rate of change of the radius of the oil meniscus in the wetting ridge (Fig. 2h). Since $\left|\left(h-R\right)\frac{d{r}_{h}}{{dt}}\right|\, \ll \, \left|\left(h+{r}_{h}\right)\frac{{dh}}{{dt}}\right|$ and ${{{{{\mathscr{l}}}}}}\, \approx \,\left(h+{r}_{h}\right)$ (see details in Supplementary Fig. 3), this simplifies to

1 $$-\frac{d{{{{{\mathscr{l}}}}}}}{{dt}}\, \approx \,\left(\frac{h+{r}_{h}}{{{{{{\mathscr{l}}}}}}}\right)\frac{{dh}}{{dt}}\, \approx \,\frac{{dh}}{{dt}}.$$

Equation (1), therefore, predicts that the magnitude of the droplet approach velocity ($-d{{{{{\mathscr{l}}}}}}{{{{{\mathscr{/}}}}}}{dt}$) is approximately equal to the oil rise velocity (${dh}/{dt}$), which we observe experimentally in Fig. 2f, g. The peak velocities ${u}_{1}\, \approx \,{\left(-d{{{{{\mathscr{l}}}}}}{{{{{\mathscr{/}}}}}}{dt}\right)}_{\max }$ and ${u}_{h}\, \approx \,{\left({dh}/{dt}\right)}_{\max }$ are also equal, i.e., ${u}_{1}\, \approx \,{u}_{h}$ ≈ 4 cm/s. Experimentally, we found that *u*_1_ decreases with ${h}_{{wr}}/R$ (Supplementary Fig. 4), where ${h}_{{wr}}$ is the height of the wetting ridge on the non-interacting side of the droplet (Fig. 2a). Further simplification of Eq. (1) by taking the second time derivative also shows that the acceleration at which the droplets approach each other ($-{d}^{2}{{{{{\mathscr{l}}}}}}{{{{{\mathscr{/}}}}}}d{t}^{2}$) is approximately equal in magnitude with the acceleration of the oil rise in the wetting ridge (${d}^{2}h/d{t}^{2}$) (Supplementary Fig. 5). Importantly, in our experiments, the relation ${\left(-d{{{{{\mathscr{l}}}}}}{{{{{\mathscr{/}}}}}}{dt}\right)}_{\max }$ $\approx$ ${\left({dh}/{dt}\right)}_{\max }$ remains true (Fig. 2i) irrespective of droplet volume (2–10 µl), oil viscosity (5–100 cP), micropillar dimensions (pillar diameter ≈5–10 µm, spacing ≈10–50 µm, and height ≈10–30 µm, Supplementary Fig. 1), and lubrication film thickness (20–30 µm) measured using white-light interferometry (Supplementary Fig. 6).

We rationalize the magnitude of *u*_1_ between the droplets (and by extension ${u}_{h}$) by looking at the forces acting on each droplet. There is an attractive capillary force ${F}_{\gamma }\sim R{\gamma }_{o}$ between the droplets, which is balanced by the viscous force ${F}_{\eta } \sim 2.6\left(2\pi \right){\gamma }_{{ow}}R{\left({u}_{1}{\eta }_{o}/{\gamma }_{{ow}}\right)}^{2/3}$ in the lubricant film^28,47,48^, where ${\gamma }_{o}$ ≈ 19 mN/m is the surface tension of oil and ${\gamma }_{{ow}}$ ≈ 36 mN/m is the oil-water interfacial tension measured using the pendant drop method (Supplementary Fig. 7)^49^. This translates to

2 $${u}_{1}\, \approx \,{\left(\frac{{{{\upgamma }}}_{o}}{5.2{{\, \uppi }}\,{{{\upgamma }}}_{{ow}}}\right)}^{3/2}\frac{{{{\upgamma }}}_{{ow}}}{{\eta }_{o}},$$

where ${\left(\frac{{{{\upgamma }}}_{o}}{5.2{{\,\uppi }}\,{{{\upgamma }}}_{{ow}}}\right)}^{3/2}\, \approx \, 0.006$ and ${{{\upgamma }}}_{{ow}}/{\eta }_{o}$ ≈ 4 m/s for ${\eta }_{o}=$ 10 cP is the speed due to viscocapillary effects. Equation (2) predicts that ${u}_{1}\, \approx \,2 \, {{\mathrm{cm/s}}}$ which is the same order of magnitude as the observed value of 4 cm/s (Fig. 2f). Equation (2) also predicts that ${u}_{1}\propto 1/{\eta }_{o}$, which agrees reasonably well with our experiments shown in (Fig. 2j). Note that while Eq. (2) correctly captures the physical origin of ${u}_{1}$ (viscocapillary), it is only an approximation since ${F}_{\gamma }$ depends on the exact meniscus and contact line geometry, the droplet radius *R*, and wetting ridge size ${h}_{{wr}}$. Equation (2) also explains the magnitude of the collision time, since *t* ≈ ${{{{{\mathcal{l}}}}}}_{{{\mathrm{init}}}}/{u}_{1} \, \approx \,70\,{{{\mathrm{ms}}}}$, which is close to the experimentally observed value of 110 ms.

We also observed oscillations in the droplet position ${{{{{\mathcal{l}}}}}}$ and speed $d{{{{{\mathscr{l}}}}}}{{{{{\mathscr{/}}}}}}{dt}$ with a timescale ${\tau }_{1}$ = 20 ms for $R$ ≈ 1 mm (Fig. 2f). The oscillation of the droplet can be modeled as an underdamped mass-spring system (Supplementary Movie 1) with mass $m \sim {\rho R}^{3}$ (where $\rho$ is the density of water) and the effective surface tension ${\gamma }_{{eff}}$ playing the role of a spring. Hence, we expect ${\tau }_{1} \sim {(\rho {R}^{3}/{\gamma }_{{eff}})}^{1/2}$, which we verified experimentally for droplets of different $R$ ≈ 0.9–1.7 mm (Fig. 2k). Note that since silicone oil is known to encapsulate water droplets^31,44^, the effective surface tension is ${\gamma }_{{eff}}={\gamma }_{o}+{\gamma }_{{ow}}$ to account for the presence of two interfaces, namely the oil-air (${\gamma }_{o}$) and oil-water (${\gamma }_{{ow}}$) interfaces (See schematic in Fig. 2a). Finally, the lubricant oil dissipates the kinetic energy, playing the role of a damping coefficient, a point that we will discuss later in the manuscript.

### Wetting ridge oil drainage: time-lag $\triangle {{{{{\boldsymbol{t}}}}}}$ (stage II)

**Fig. 3:**
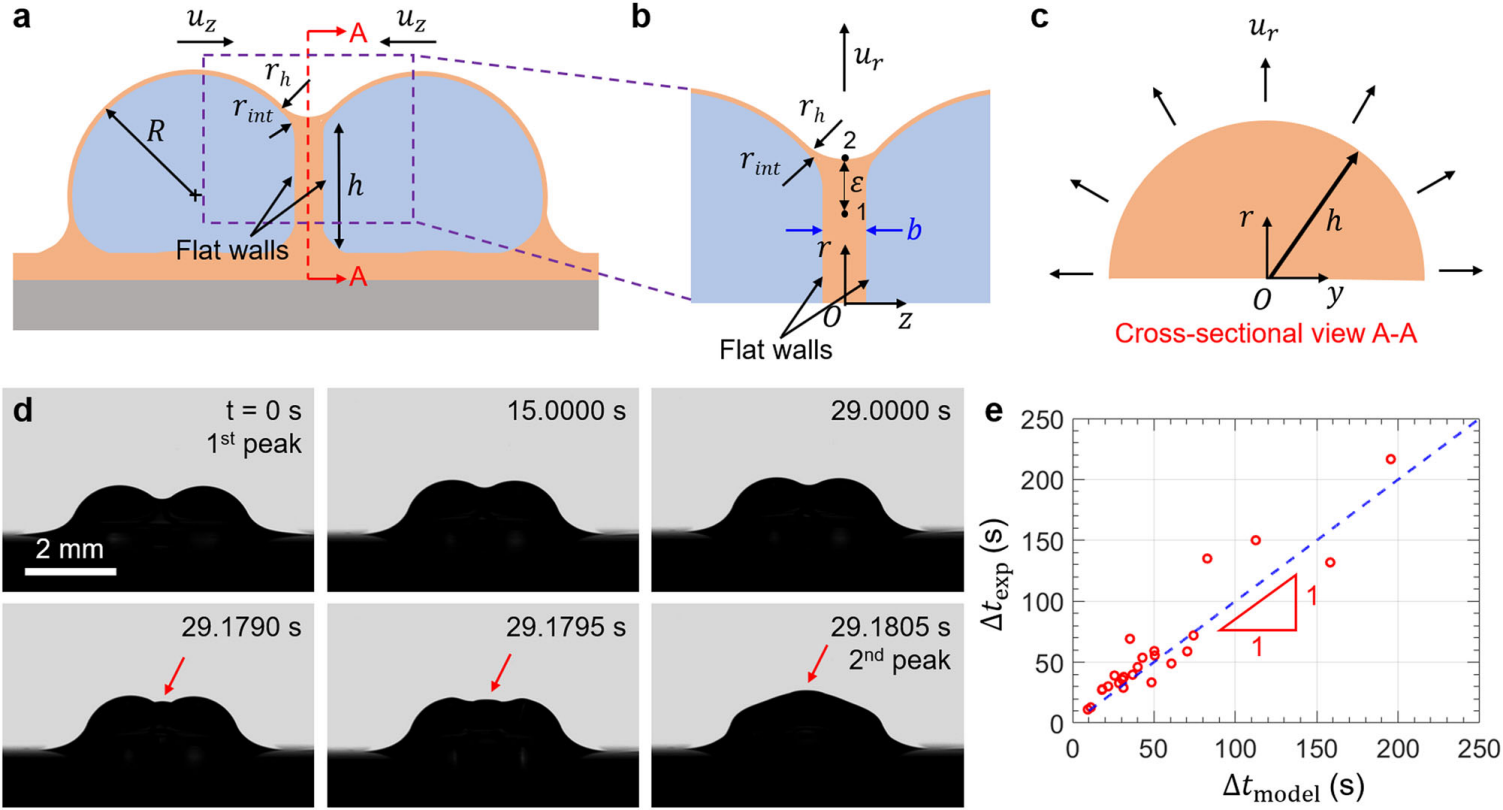
Droplet coalescence model. **a** Schematic of coalescing droplets. The oil between the two droplets needs to be drained before coalescence. **b** Magnified view of the mid-section of coalescing droplets showing the flat wall that forms between them. **c** Cross-sectional view A-A of the mid-section of the wetting ridge. The oil is drained radially outwards with velocity ${u}_{r}$. **d** Time-lapse images of coalescing droplets. High pressure (convex meniscus indicated by arrow) builds up in the wetting ridge after the first peak. **e** The experimental time-lag (${\triangle}{t}$) agrees with the theoretical prediction (${{\triangle}t}_{{{\mathrm{model}}}}$) that is obtained by approximating the oil drainage as a flow between two parallel plates (Poiseuille flow).

After the initial attraction, the droplets collide with each other and deform substantially to form a nearly flat wall^50^ (Fig. 3a, b), whose geometries can be approximated as semicircles with radius $h \, \approx \, R$ (Fig. 3c, see exact droplet and wall geometries in Supplementary Fig. 8). A thin lubricant film of thickness $b$ and volume $0.5\pi {h}^{2}b$ is trapped between the two walls, which slowly drains out with average radial velocity ${u}_{r}$. The pressure difference driving the flow is

3 $$\triangle p={p}_{1}-{p}_{2}=\frac{2\left({\gamma }_{o}+{\gamma }_{{ow}}\right)}{R}-\frac{{\gamma }_{o}}{h}+\frac{{\gamma }_{o}}{{r}_{h}}.$$

where *p*_1_ and *p*_2_ are the pressures at points 1 and 2 (Fig. 3b), respectively.

We follow the analysis by Landau and Levich and apply the lubrication approximation to the flow in the transition region of size $\varepsilon \sim {\left(b{r}_{h}\right)}^{1/2}$ joining the flat film and the annular meniscus region (Fig. 3b)^51^. According to the Poiseuille model, the radial velocity of oil drainage is ${u}_{r} \sim \nabla p ({(b/2)}^{2}-{z}^{2})/{\eta }_{o}$, where $\nabla p \sim \triangle p/\varepsilon$ is the radial pressure gradient driving the flow with $\triangle p$ given by Eq. (3). The corresponding volume flow rate is $Q \sim \pi h{\int }_{-b/2}^{b/2}{u}_{r}{dz} \sim \nabla p{b}^{3}h/{\eta }_{o}$. Since $Q \sim {h}^{2}({db}/{dt})$, we can get an expression for the rate at which the lubricant film thickness decreases with time ${db}/{dt} \sim \nabla p{b}^{3}/{\eta }_{o}h$, which can be integrated to find the time required to fully drain the oil in the wetting ridge between the droplets as

4 $${\triangle t}_{{{{{{\rm{model}}}}}}}=\frac{{\eta }_{o}h{{r}_{h}}^{1/2}}{\triangle p}\left(\frac{1}{{{b}_{f}}^{3/2}}-\frac{1}{{{b}_{i}}^{3/2}}\right)\, \approx \,\frac{{\eta }_{o}h{{r}_{h}}^{1/2}}{\triangle p{{b}_{f}}^{3/2}},$$

where ${b}_{i}$ and ${b}_{f}$ are respectively the initial and final oil thicknesses with ${b}_{f}$ ≪ ${b}_{i}$. The final oil thickness ${b}_{f}$ refers to the condition when the oil film becomes unstable due to van der Waals interactions (in the order of 100 nm). The oil drainage process presented in this study is analogous to numerous past investigations reported in the literature^52–54^, except that the compressive force is primarily due to the capillary suction in the wetting ridge (rather than droplet weight or inertial effects). This fluid flow is analogous to drainage of a water film in foams with low-pressure regions in the Plateau borders^55^, which can be modeled as a Poiseuille flow. Though in foams, the drainage is complicated by the presence of surfactants, which are absent in this study.

The time-lapse images of the two coalescing droplets in Fig. 3d show experimental drainage time of ${\triangle t}_{{{\exp }}}$ = 29 s (equivalent to time-lag in Fig. 1). We were able to identify the point at which the oil film becomes unstable by noting the change in meniscus shape from concave to convex at time *t* > 29 s (arrows, Fig. 3d). More images of the oil draining process are shown in Supplementary Fig. 9. We find excellent agreement between ${\triangle t}_{{{\exp }}}$ and ${\triangle t}_{{{{{{\rm{model}}}}}}}$ (Eq. (4)) assuming ${b}_{f}$ = 150 nm with no other fitting parameter. The agreement is shown by the dashed line in Fig. 3e where different data points were obtained by varying the oil viscosity and wetting ridge size (and hence different $h$ and ${r}_{h}$, Fig. 2a). Our model, therefore, correctly captures the oil drainage process and rationalizes the time-lag $\triangle t$ between the two velocity and acceleration peaks.

### Droplet merging: second peak ${{{{{{\boldsymbol{u}}}}}}}_{{{{{{\boldsymbol{2}}}}}}}$ and ${{{{{{\boldsymbol{a}}}}}}}_{{{{{{\boldsymbol{2}}}}}}}$ (stage III)

**Fig. 4:**
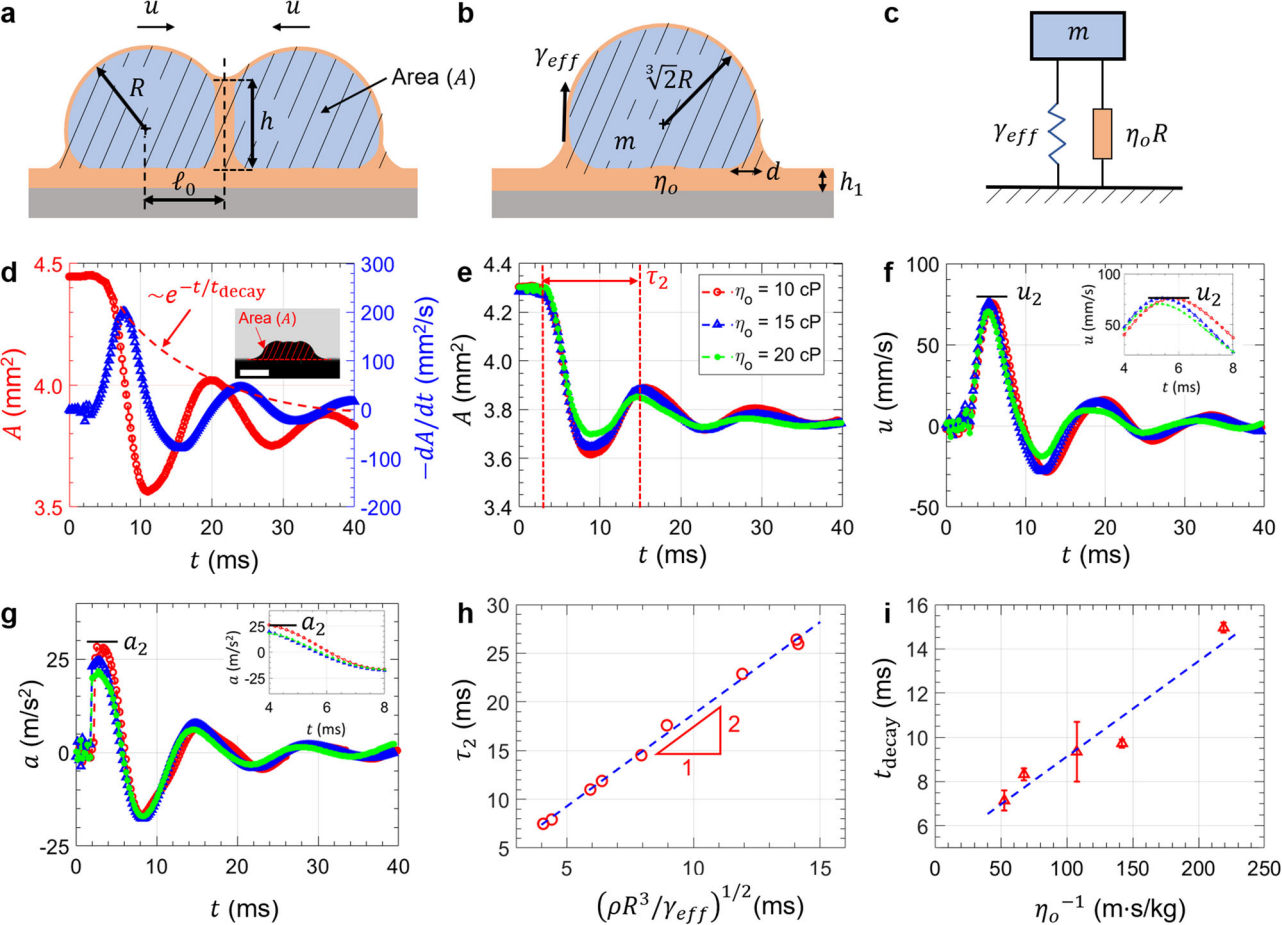
Droplet coalescence mechanism. **a** After the initial attraction, the droplets need to squeeze out the oil in the wetting ridge before coalescence. **b** Two droplets coalesce into one large droplet (${h}_{1}$ is the lubrication film thickness and $d$ is the size of the rim around the droplet base). **c** Modeling droplet coalescence using the standard mass-spring harmonic oscillator. Surface tension and oil viscosity play the roles of spring and damper, respectively. **d** Cross-sectional area (including the wetting ridge) of two coalescing droplets ($A$, left axis) and its time-derivative ($-dA/dt$, right axis) as a function of time. Both $A$ and $-dA/dt$ decay exponentially (inset scale bar = 2 mm). **e** Underdamped oscillation of the droplet for varying oil viscosity (10 cP, 15 cP, and 20 cP). The period of oscillation is nearly unaffected by oil viscosity. Velocity (**f**) and acceleration (**g**) of coalescing droplets as a function of time. **h** Period of oscillation ($\tau_{2}$) scales with ${(\,\rho {R}^{3}/{\gamma }_{{eff}})}^{1/2}$, a result that is analogous to the standard mass-spring systems with $(m/k)^{1/2}$ period, where $m$ is the mass ($m \sim {\rho} R^{3}$) and $k$ is the spring constant. **i**, In agreement with standard harmonic oscillators, the decay time ($t_{{{\mathrm{decay}}}}$) scales inversely with oil viscosity ($t_{{{\mathrm{decay}}}} \sim {\eta_{o}}^{-1}$). Error bars represent one standard deviation.

When the oil film becomes unstable, the two droplets (each with initial radius $R$ and total surface area $2\times2\pi {R}^{2}=4\pi {R}^{2}$) merge into a single droplet with a new radius ${R{{\hbox{'}}}}$ = 2^1/3^$R$ (from conservation of mass) and a smaller total surface area ${2}^{5/3}\pi {R}^{2}$ (Fig. 4a, b; see also Supplementary Movie 2). The release of free surface energy during the droplet merging, which can be modeled using a standard mass-spring system (Fig. 4c–e), is converted into translational kinetic energy and hence explains the magnitude of the second velocity peak ${u}_{2}$ in stage III, i.e.,

5 $$2\left[\frac{1}{2}\left(\frac{2}{3}\rho \pi {R}^{3}\right){{u}_{2}}^{2}\right]	\, \approx \,(4-{2}^{5/3})\pi {R}^{2}{\gamma }_{{eff}},\\ {u}_{2}	\, \approx \, 1.1{\left(\frac{{\gamma }_{{eff}}}{\rho R}\right)}^{\frac{1}{2}}.$$

Hence, for millimeter-size droplets, we expect ${u}_{2}$ ≈ 20 cm/s if all of the interfacial energy liberated from coalescence is converted to in-plane kinetic energy. Experimentally, we measured substantially smaller ${u}_{2}$ ≈ 8 cm/s (Fig. 4f) which suggests that only a small fraction of the interfacial energy (≈ 16%) is converted to kinetic energy. Importantly, Eq. (5) predicts that ${u}_{2}$ (unlike ${u}_{1}$) is independent of ${\eta }_{o}$ (cf. Equation (2)). In our measurements, the second peak acceleration ${a}_{2}$ ≈ 25 m/s^2^ (Fig. 4g) is larger than the first peak (${a}_{1}$ ≈ 5 m/s^2^, Fig. 1d). Additionally, we estimated ${u}_{2}$ ≈ 14 cm/s by tracking the intersection point between the wetting ridge and the droplet (see Supplementary Fig. 10 and Movie 3). This method, however, overestimates ${u}_{2}$ since the intersection point continues to move in the horizontal direction after the center of mass of the droplet becomes stationary (that is, the droplet elongates in the vertical direction).

For droplet coalescence on traditional lotus-leaf-inspired air-filled superhydrophobic surfaces, the release of interfacial energy also results in out-of-plane droplet jumping due to minimal contact line pinning^56^. On textured oil-impregnated surfaces, however, droplet jumping is suppressed because of strong capillary adhesion to the wetting ridge, which confined the translational kinetic energy to in-plane motion. Note that Eq. (5) is reminiscent of a mass-spring system with mass $m \sim \rho {R}^{3}$ and ${\gamma }_{{eff}}$ playing the role of the spring constant. Therefore, we expect the droplets to oscillate with a typical timescale ${\tau }_{2} \sim {(\, \rho {R}^{3}/{\gamma }_{{eff}})}^{1/2}$. There is also viscous dissipation in the lubricant oil with ${\eta }_{o}$ playing the role of damping (schematic in Fig. 4c). However, we will show later that the mass-spring system is underdamped, and ${\eta }_{o}$ does not substantially affect ${u}_{2}$ or ${\tau }_{2}$ (See a more detailed analysis of the mass-spring analogy in Supplementary Discussion Section S7).

Since in stage III, the droplets can no longer be approximated as hemispheres due to substantial deformation, we estimated the droplet velocity $u$ and acceleration $a$ by measuring the cross-sectional area *A* (shaded region in Fig. 4a, b) and using relations $u={-{0.5}}({dA}/{dt})/h$ and $a={-{0.5}}({d}^{2}A/d{t}^{2})/h$. The 0.5 factor is included in the calculation since $A$ is the total cross-sectional area of both droplets while the meniscus height $h$ is used to convert area into length. Experimentally, we found that *A* (left axis, Fig. 4d) and $-{dA}/{dt}$ (right axis, Fig. 4d) oscillate with period ${\tau }_{2}$ with an exponential decay envelope ${e}^{-t/{t}_{{{{{{\rm{decay}}}}}}}}$, where ${t}_{{{{{{\rm{decay}}}}}}}$ is a characteristic decay time (Fig. 4d). While the oscillation amplitude and the decay envelope differ for different ${\eta }_{o}$ = 10 cP, 15 cP, and 20 cP, ${\tau }_{2}$ is independent of ${\eta }_{o}$ (Fig. 4e). We also found that ${u}_{2}$ and ${a}_{2}$ are independent of ${\eta }_{o}$, consistent with Eq. (5) (Fig. 4f, g). Experimentally, we proved that ${{\tau }_{2} \sim (\,\rho {R}^{3}/{\gamma }_{{eff}})}^{1/2}$ (Fig. 4h), the same scaling law with the oscillation period ${\tau }_{1}$ in stage I (Fig. 2k). These two scaling laws show that the period of oscillation at both the first and second peaks scales with droplet radius to the 3/2 power, i.e., ${\tau }_{2}\propto {R}^{3/2}$. We also experimentally showed that ${t}_{{{{{{\rm{decay}}}}}}}\propto {{\eta }_{o}}^{-1}$ (Fig. 4i). The scaling laws for both oscillation period and decay time are consistent with an underdamped mass-spring harmonic oscillator with ${\gamma }_{{eff}}$ and ${\eta }_{o}$ playing the role of the spring constant and damping coefficient, respectively.

In summary, we show that droplet-droplet interaction on textured oil-impregnated surfaces is qualitatively different from droplet-droplet interaction on traditional lotus-leaf-inspired superhydrophobic surfaces due to the presence of wetting ridge that forms near the droplet base due to the imbalance of interfacial forces at the contact line. On micro/nanotextured oil-impregnated surfaces, water droplets interact with one another remotely through their wetting ridge and coalesce in three distinct stages: attraction between the droplets (stage I), drainage of the oil in the wetting ridge (stage II), and coalescence or merging (stage III). Analysis of high-speed images captured at 5000 fps shows that stage I (attraction) and stage III (coalescence) leave their signature as velocity (${u}_{1}$ and ${u}_{2}$) and acceleration (${a}_{1}$ and ${a}_{2}$) peaks, while stage II appears as a time-lag $\triangle t$ between the velocity and acceleration peaks. Experiments show that the horizontal velocity (${u}_{1}$ ≈ 4 cm/s) and acceleration (${a}_{1}$ ≈ 5 m/s^2^) at which the droplets approach each other at the first peak are equal in magnitude to the vertical oil rise velocity and acceleration in the wetting ridge. This is the first report that shows such a unique feature. We captured this important physics that is independent of droplet size, oil viscosity, micropillar dimensions, and lubricant film thickness by developing a simple geometry-based model and scaling analysis that agrees well with experiments and high-speed visualization. The second peak velocity (${u}_{2}$ ≈ 8 cm/s) and acceleration (${a}_{2}$ ≈ 25 m/s^2^) due to the release of free surface energy (i.e., reduction in surface area) are slightly higher than the first peak. We model droplet coalescence using a standard mass-spring harmonic oscillator where surface tension and oil viscosity play the respective roles of a spring constant and damping coefficient. This model, which is validated using high-speed visualization and experiments, shows that the period of oscillation at the first and second peaks scales with droplet radius to the 3/2 power, i.e., $\tau \sim {(\, \rho {R}^{3}/{\gamma }_{{eff}})}^{1/2}$. Unlike the first velocity peak, which scales inversely with oil viscosity (${u}_{1}\propto 1/{\eta }_{o}$), the second velocity peak (${u}_{2}$) does not depend on oil viscosity. Experiments show that the time-lag $\triangle t$ (that is, the time required to fully drain the oil in the wetting ridge before coalescence) varies from 10 to 200 s depending on the oil viscosity and wetting ridge volume. Our model for the time-lag, which agrees with experiments, captures the fundamental physics governing the process by approximating the oil drainage using a Poiseuille flow model between parallel plates. This work provides a fundamental understanding of droplet-droplet interaction and coalescence mechanism on textured oil-impregnated surfaces. The mechanistic insights gained from this work have a potential to further manipulate droplets to improve the overall efficiency of important engineering processes such as water/fog harvesting and condensation heat transfer.

## Methods

### Materials

Silicone oil and Trichloro(1H,1H,2H,2H-perfluorooctyl) silane were supplied by Sigma-Aldrich. All chemicals were used as-is without further purification.

### Sample fabrication

We fabricated well-controlled silicon micropillars using contact photolithography and deep reactive ion etching (DRIE). The test samples (silicon micropillars) were plasma treated (PDC-001-HP, Harrick Plasma) for 30 min and silanized using trichloro(1H,1H,2H,2H-perfluorooctyl) silane in a desiccator. Scanning electron microscopy (SEM) images of the silicon micropillars before oil impregnation are provided in Supplementary Fig. 1. Following silanization, the silicon micropillars were impregnated with chemically compatible silicone oil of the desired viscosity.

### Droplet placement

Two millimeter-size water droplets were placed on the oil-impregnated silicon micropillar surface that was securely attached to an x-y-z stage (LT3-XYZ Translation Stage, Thorlabs). One of the droplets was attached to a 200 µm diameter capillary tube (HR6-104, Hampton Research) using the meniscus that forms when the glass surface touches the droplet. The droplets were brought close to each other in 10 µm increments until they eventually attract each other by exerting force remotely through the wetting ridge. When the droplets start to move toward each other, the capillary tube was pulled out vertically to minimize its interference on the droplet coalescence mechanism.

### Droplet tracking

The droplet interaction and the resulting motion was captured by acquiring images at 5000 fps using a high-speed camera (Phantom v1610, Vision Research) and magnifying lens (AF NIKKOR 50 mm f/1.4 G, Nikon). The lens was operated at $f/1.4$ and with magnification $M=-2.6$ corresponding to an object-plane pixel size of 10.9 µm. The spatial resolution of the camera-lens combination was measured to be 28 µm based on the 50% cut-off frequency of the modulation transfer function measured using the slanted knife-edge test. The camera integration duration was fixed at 3 µs. Droplets were backlit using a white LED (Fiber-Lite MI-150, Dolan-Jenner). The time-lapse images were analyzed using MATLAB by fitting a circle (i.e., circular Hough transform) around the droplet and a circular arc on the oil meniscus in the wetting ridge. We transform the image to binary by using rgbtogray function and use ‘Canny’ edge detection method in MATLAB to detect the droplet shape. Due to substantial shape distortion, the cross-sectional area of the droplet (instead of circle fitting) was used for analyzing the second peak.

### Film thickness measurement

We used white-light interferometry technique to measure the oil film thickness^57^. The experimental setup consists of a reflection probe (RP21, ThorLabs), a pocket spectrometer (FLAME-S-VIS-NIR, Ocean Insight), and a broadband quartz-tungsten halogen lamp (HL-2000-LL, Ocean Insight). Film thickness measurement is discussed in detail in Supplementary Discussion Section S4.

### Supplementary information

Supplementary Information

Description of Additional Supplementary Files

Supplementary Movie 1

Supplementary Movie 2

Supplementary Movie 3

## Data Availability

All data are available, either in numerical or graphical form, in the main text or the Supplementary Information.
