# Supplementary material for: Droplet attraction and coalescence mechanism on textured oil-impregnated surfaces
Source: Nat Commun. 2023 Aug 18;14:4901. doi: 10.1038/s41467-023-40279-w (PMC10439220; doi:10.1038/s41467-023-40279-w)
Supplement: Supplementary file 1 — Supplementary Information [file 41467_2023_40279_MOESM1_ESM.pdf]

Droplet attraction and coalescence mechanism on textured oil-impregnated surfaces

Haobo Xu[1], Yimin Zhou[1], Dan Daniel[2], Joshua Herzog[1], Xiaoguang Wang[3,4], Volker Sick[1], Solomon Adera[1,*]
[1]Department of Mechanical Engineering, University of Michigan, Ann Arbor, MI, USA
[2]Division of Physical Sciences and Engineering, King Abdullah University of Science and Technology (KAUST), Thuwal, Saudi Arabia
[3]Department of Chemical and Biomolecular Engineering, The Ohio State University, Columbus, OH, USA

[4]Sustainability Institute, The Ohio State University, Columbus, OH, USA

*Corresponding author: Solomon Adera
Email: sadera@umich.edu

Content

S1: Test sample fabrication

S2: Droplet shape

S3: Droplet approaching velocity and acceleration

S4: Film thickness measurement

S5: Interfacial force measurement using the pendant drop method

S6: Droplet shape during oil drainage

S7. Coalescence-induced droplet harmonic oscillation

S8. Measuring second velocity peak by tracking wetting ridge-droplet intersection point

S1. Test sample fabrication

Well-controlled silicon micropillars were fabricated in a square pattern using the standard silicon fabrication technology (contact photolithography and deep reactive ion etching). The micropillar diameter (d_p≈ 5-10 μm), spacing/pitch (p ≈10-50 μm), and height (h_p ≈10-30 μm) were varied during fabrication. The silicon micropillars were silanized and impregnated with silicone oil of varying viscosity η_o = 5-100 cP. The lubrication film thickness was varied between h_ℓ ≈20-30 μm by controlling the rotational speed on the spin coater (H6-23, Laurell Technologies). Scanning electron microscope (SEM) images at different magnifications at 10° inclination angle before oil impregnation are shown in Supplementary Fig. 1.

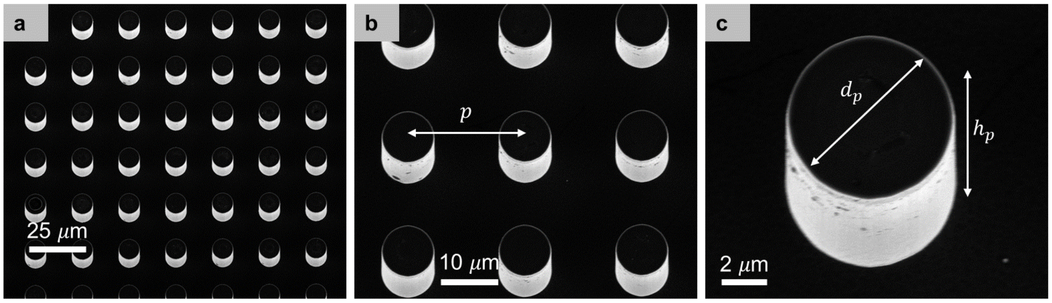

Supplementary Figure 1. **Scanning electron microscopy (SEM). a-c** SEM images at 10° inclination of cylindrical silicon micropillars at different magnifications before oil impregnation. The diameter (d_p), spacing/pitch (p), and height (h_p) of the micropillars were varied during fabrication.

S2: Droplet shape

A water droplet (Supplementary Fig. 2a) was deposited on an oil-impregnated silicon micropillar surface. Images of the droplet were captured using the built-in camera of a drop shape analyzer (DSA100E, KRÜSS GmbH) and analyzed using MATLAB. Irrespective of variations in droplet volume, oil viscosity, lubrication film thickness, and silicon micropillar diameter, spacing, and height, the droplet radius R and base radius R_b are nearly equal as shown in Supplementary Fig. 2b. Moreover, the volume-equivalent droplet radius (R^*), which is obtained from the volume (Ω) assuming a hemisphere as $R^* = (3\Omega/2\pi)^{1/3}$, is approximately equal to the droplet radius (R) obtained from image analysis (Supplementary Fig. 2c). Both measurements in Supplementary Fig. 2b,c show that water droplets on oil-impregnated surfaces are nearly hemispherical with ≈90° apparent contact angle.

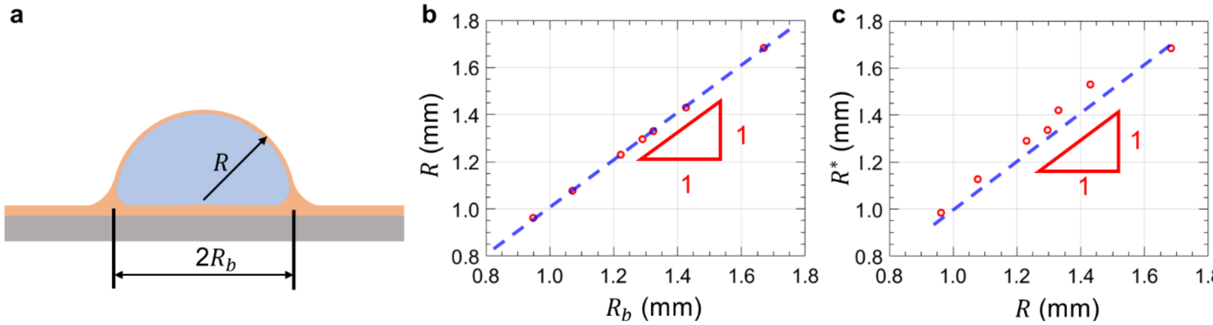

Supplementary Figure 2. **Droplet shape. a** A sessile droplet residing on an oil-impregnated surface. **b** Droplet radius R and base radius R_b are nearly equal. **c** Volume equivalent droplet radius (R^*) and actual droplet radius from image (R) are nearly equal.

S3: Droplet approaching velocity and acceleration

As the two droplets approach each other (Supplementary Fig. 3a), they deform substantially. Particularly, the side where the droplets face each other becomes nearly flat with a thin film of oil sandwiched between the droplets as shown in Supplementary Fig. 3b.

Comparing the terms on the left-hand side of Equation (1) in the main text by analyzing the high-speed videos shows that $\delta \ll \beta$, where $\delta = (h - R)\frac{dr_h}{dt}$ and $\beta = (h - R)\frac{dr_h}{dt} + (h + r_h)\frac{dh}{dt}$ (Supplementary Fig. 3c). Furthermore, our experiments show that the coefficients of dh/dt and $-d\ell/dt$ in Equation (1) are comparable near the first peak, that is, $h + r_h \approx \ell$ (Supplementary Fig. 3d). Making use of these two approximations simplifies Equation (1) to $-d\ell/dt = dh/dt$, which implies that the horizontal velocity at which the droplets approach each other ($-d\ell/dt$) is equal in magnitude to the vertical oil rise velocity in the wetting ridge (dh/dt). The model derived from geometry alone agrees well with our experiment.

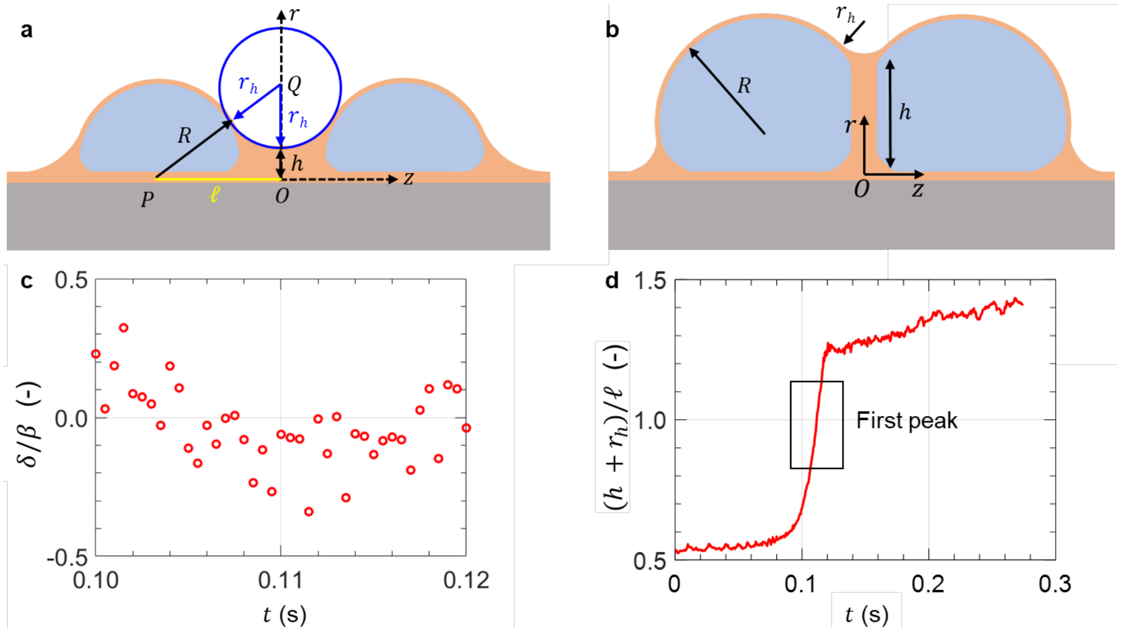

Supplementary Figure 3. **First peak velocity approximation. a,b** Pre-coalescence droplet attraction. The centers of the droplet (P), oil meniscus (Q), and wetting ridge (O) form a right triangle. **c** The first term in Equation (1) is small compared to the left-hand side ($\delta/\beta \ll 1$), suggesting it can be dropped from the equation. **d** Near the first peak, experiments show that $h + r_h \approx \ell$. This allows further simplification of Equation (1) to $-d\ell/dt = dh/dt$, that is, the velocity at which the droplets approach each other and the oil rise velocity are equal in magnitude.

High-speed image analysis by fitting droplets using a circle in MATLAB shows that the peak velocity during attraction increases with droplet volume Ω (Supplementary Fig. 4a) while it decreases with the ratio h_{wr}/R (Supplementary Fig. 4b), where R is the droplet radius and h_{wr} is the height of the wetting ridge (Supplementary Fig. 3a). Note that h_{wr}/R indicates the relative volume of oil that needs to be pushed out by the droplets as they approach each other.

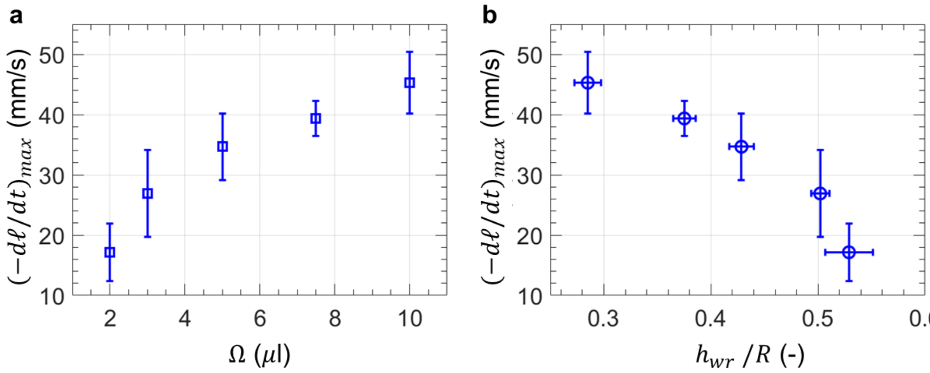

Supplementary Figure 4. **First peak velocity. a** Approach velocity increases with droplet volume (Ω). **b** Approach velocity decreases with h_{wr}/R ratio, where h_{wr} is the height of the wetting ridge and R is the droplet radius. The ratio

h_{wr}/R is the relative measure of the volume of oil that puts up resistance to droplet motion. Error bars represent standard deviation.

Taking the derivative with respect to time of Equation (1) gives

$$-\left(\frac{d\ell}{dt}\right)^2 - \ell\frac{d^2\ell}{dt^2} = 2\frac{dh}{dt}\frac{dr_h}{dt} + (h-R)\frac{d^2r_h}{dt^2} + \left(\frac{dh}{dt}\right)^2 + (h+r_h)\frac{d^2h}{dt^2}. \quad (1)$$

The terms $\frac{dh}{dt}\frac{dr_h}{dt}$, $(h-R)\frac{d^2r_h}{dt^2}$, $\left(\frac{dh}{dt}\right)^2$, and $\left(\frac{d\ell}{dt}\right)^2$ are in the order of 10^{-4} while the other two terms $(h+r_h)\frac{d^2h}{dt^2}$ and $\ell\frac{d^2\ell}{dt^2}$ are in the order of 10^{-2}. This in combination with $h + r_h \approx \ell$ is used to simplify Supplementary Equation (1) to $-d^2\ell/dt^2 = d^2h/dt^2$, which shows that the acceleration at which the droplets approach each other horizontally and the vertical oil rise acceleration are equal in magnitude. This outcome from geometric modeling is validated experimentally (Supplementary Fig. 5).

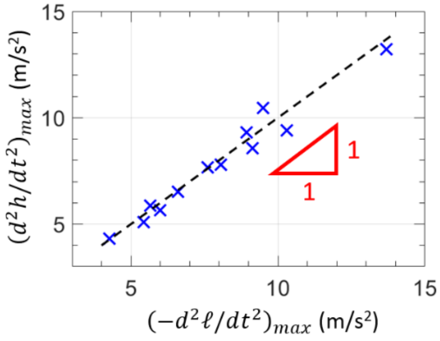

Supplementary Figure 5. **First peak acceleration.** The horizontal acceleration at which the two droplets approach each other ($-d^2\ell/dt^2$) and the vertical acceleration of oil rise in the wetting ridge (d^2h/dt^2) are equal in magnitude.

S4. Film thickness measurement

Thickness of the oil used for impregnation was measured via white-light interferometry. Briefly, a reflection probe (RP21, ThorLabs) coupled to a pocket spectrometer (FLAME-S-VIS-NIR, Ocean Insight) and broadband quartz-tungsten halogen lamp (HL-2000-LL, Ocean Insight) was used to measure reflection spectra (Supplementary Fig. 6) from the oil-impregnated silicon pillars. The probe tip was placed approximately 2 mm above the surface, and the spectrometer was integrated for 5 ms to measure the reflected QTH lamp spectrum. A diffuse reflection standard (WS-1, Ocean Insight) was measured in an identical fashion to produce a reference spectrum such that the reflection coefficient can be calculated as

$$R(\lambda) = \frac{I(\lambda) - B(\lambda)}{I_{\mathrm{ref}}(\lambda) - B_{\mathrm{ref}}(\lambda)}, \quad (2)$$

where I is the measured intensity, B is the background measurement (taken with the QTH lamp shutter closed), λ is the wavelength, and the subscript ref refers to the measurement of the reference sample. The film thickness is determined from the reflection spectrum using the following theory.

Assuming the oil-air and oil-substrate interfaces are perfectly flat (neglecting the step change due to the microstructures), the reflection coefficient of any point at normal incidence is given by

$$R(\lambda) = \frac{r_{ao}^2 + r_{os}^2 + 2r_{ao}r_{os}\cos\left(\frac{4\pi n_o b}{\lambda}\right)}{1 + r_{ao}^2 r_{os}^2 + 2r_{ao}r_{os}\cos\left(\frac{4\pi n_o b}{\lambda}\right)}, \quad (3)$$

where r_{ao} and r_{os} are the Fresnel amplitude-reflection coefficients of the air-oil and oil-substrate interfaces, respectively, for either p- or s-polarization (in general, R is the weighted average of the two individual polarizations), and n_o is the refractive index of the oil film. The oil film thickness is given by b. Typically,

$r_{ao}^2 \ll 1$ (for $n_o = 1.41, r_{ao}^2 = 0.04$). Reflection at the oil-substrate interface is larger but still small compared to unity, so the reflection coefficient is approximately

$$R(\lambda) \approx r_{os}^2 + 2r_{ao}r_{os}\cos\left(\frac{4\pi n_o b}{\lambda}\right) \approx c_1 + c_2\cos(2kn_o d), \tag{4}$$

where k is the vacuum wave number and c_1 and c_2 are slowly varying due to the wavelength dependence of the refractive indices.

To calculate film thickness, the measured reflection spectrum is re-sampled into k-space to determine $R(k)$, and a detrending operation is performed (subtraction of a low-order polynomial fit) to remove the slowly-varying contribution from the wavelength-dependence of c_1 and c_2. The discrete Fourier transform of the filtered $R(k)$ is calculated, and a Cauchy distribution is fit to the peak of the spectrum. The location of the peak corresponds to the mean value $2n_o b$, from which the mean value of b is determined. The width of the peak provides a measurement of uncertainty, partially arising from wavelength dependence of n_o. As shown in Supplementary Fig. 6, we analyzed the data and obtained two thickness values, b_1 and b_2 are 11 and 30 μm, respectively. We use b_2 as the thickness of oil film since the oil film thickness is larger or equal to the height of the micropillars ($h_p \approx 20$ μm).

For microstructured surfaces with fully submerged micropillars, reflections from the top of the pillars as well as from the pillar base (that is, the shallow region between the pillars) produce interference effects that are visible in the reflection spectra as a superposition of cosine components. This produces a bi-modal distribution in the Fourier transform of $R(k)$, which is captured well by the analysis procedure presented here, showing two peaks in the Fourier spectrum with relative intensities determined by the micropillar area fraction. In principle, fitting both peaks provides a measure of the areal film-thickness distribution. Note that the reflection spectrum is intensity-weighted, and so the relative intensity of the Fourier-transform peaks may be biased as a result of probe size and positioning.

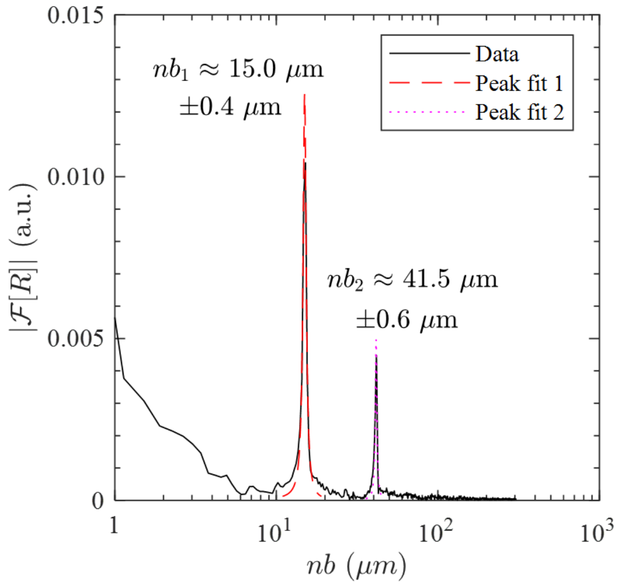

Supplementary Figure 6. **Film thickness.** Calculated Fourier-amplitude spectrum of $R(k)$ measured on a patterned substrate showing two distinct peaks. Assuming the average oil refractive index is approximately 1.41, b_1 and b_2 are 11 and 30 μm, respectively.

S5: Interfacial force measurement using the pendant drop method

We measured the surface tension of silicone oil by using the pendant drop method. Briefly, a silicone oil droplet is pushed out of a stainless-steel needle slowly at 0.05 μl/s to minimize dynamic effects of fluid motion, and images were captured at 30 fps (Supplementary Fig. 7a). The interfacial tension (IFT) was

calculated by balancing the buoyant force on the near-departure droplet with the surface tension force around the needle tip using the built-in software of the drop shape analyzer (DSA100E, KRÜSS GmbH). Based on our measurement, the oil-air interfacial tension of silicone oil is 18.57 mN/m. Since silicone oil (930 kg/m^3) is lighter than water (1000 kg/m^3), we dispensed a silicone oil droplet inside a water bath (Supplementary Fig. 7b) to take advantage of buoyancy. This was done using a stainless-steel J-type needle (Supplementary Fig. 7c). This measurement shows that the oil-water interfacial tension of silicone oil is 35.99 mN/m. We also measured the surface tension of water in air environment (72.18 mN/m). The spreading coefficient of silicone oil on water, which is derived by minimizing the surface free energy, is given by

$$S_{ow(v)} = \gamma_{wa} - \gamma_{oa} - \gamma_{ow}, \tag{5}$$

where $S_{ow(v)}$ is the spreading coefficient of oil on water in the presence of vapor and γ is the interfacial tension between the two phases indicated by the subscripts o, w, and a for oil, water, and air, respectively. Based on our measurements, the spreading coefficient of silicone oil on water is 17.62 mN/m; a positive spreading coefficient that indicates the presence of a wrapping layer.

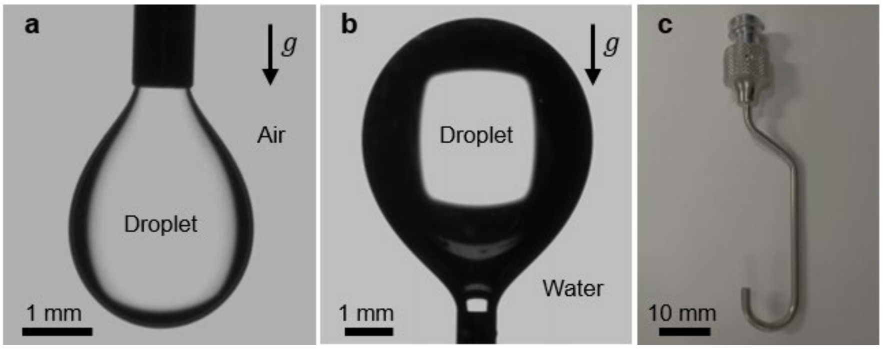

Supplementary Figure 7. **Pendant drop method for measuring interfacial tension.** **a** A silicone oil droplet suspended in air from a stainless-steel needle. **b** A silicone oil droplet (930 kg/m^3) pushed out of a J-needle where the surrounding environment is de-ionized water (1000 kg/m^3) and the density difference (buoyancy) causes the silicone droplet to rise vertically against gravity. **c** Stainless steel J-needle used for measuring the oil-water interfacial tension.

S6: Droplet shape during oil drainage

The squishy droplets deform and form a nearly flat wall that allows the oil between the droplets to drain in the radial direction. We assume that the oil drainage process is a quasi-static process with a stable droplet geometry. We modeled the thin flat oil film between the droplets as a half cylinder with a nearly constant radius $r \approx h$. A schematic side view and top view of the droplets is shown in Supplementary Fig. 8a,b. Based on these assumptions, the apparent contact angles, which describe the droplet shape, can be theoretically derived from interfacial force balance. To validate these assumptions with experiments, we captured images of coalescing droplets from the side (Supplementary Fig. 8c) and from the top (Supplementary Fig. 8d) and measured the apparent contact angles.

Theoretically, the apparent contact angle near the droplet base ($\theta_{app,1}$) can be obtained by balancing forces as

$$\cos\theta_{app,1} = \frac{\gamma_o - \gamma_{ow}}{\gamma_o + \gamma_{ow}}, \tag{6}$$

where γ_o is the surface tension of oil and γ_{ow} is the oil-water interfacial tension. Using the pendant drop method, we measured these two interfacial tensions as $\gamma_o \approx 18.6$ mN/m and $\gamma_{ow} \approx 36.0$ mN/m. The apparent contact angle estimated using Supplementary Equation (6) ($\theta_{app,1,theory}$=108°) is ≈6% higher than our measurement ($\theta_{app,1,exp}$=101°) which involves image analysis using MATLAB.

Additionally, we estimated the apparent contact angle at the meniscus top ($\theta_{app,2}$) using

$$\cos\theta_{app,2} = \cos\theta_{app,3} = -\frac{\gamma_{ow}}{\gamma_o + \gamma_{ow}}. \quad (7)$$

Our measurements for $\theta_{app,2}$ and $\theta_{app,3}$ from image analysis using MATLAB ($\theta_{app,2,exp}$=131° and $\theta_{app,3,exp}$=138°, Supplementary Fig. 8c,d) match within $\pm 6\%$ error with the angle calculated using Supplementary Equation (7) ($\theta_{app,2,theory}$=$\theta_{app,3,theory}$=131°). These measurements are summarized in Supplementary Table 1.

Supplementary Table 1. **Apparent contact angle.** Interfacial tensions were measured using the pendant drop method and contact angles are calculated by analyzing images using curve fitting in MATLAB. Interfacial forces (γ_o, γ_{ow}) are in mN/m while angles are in degree (°).

Oil type	γ_o	γ_{ow}	$\theta_{app,1,theory}$	$\theta_{app,1,exp}$	$\theta_{app,2,theory}$	$\theta_{app,2,exp}$	$\theta_{app,3,theory}$	$\theta_{app,3,exp}$
Silicone oil, η_o = 10 cP	18.57	35.99	108	101	131	131	131	138

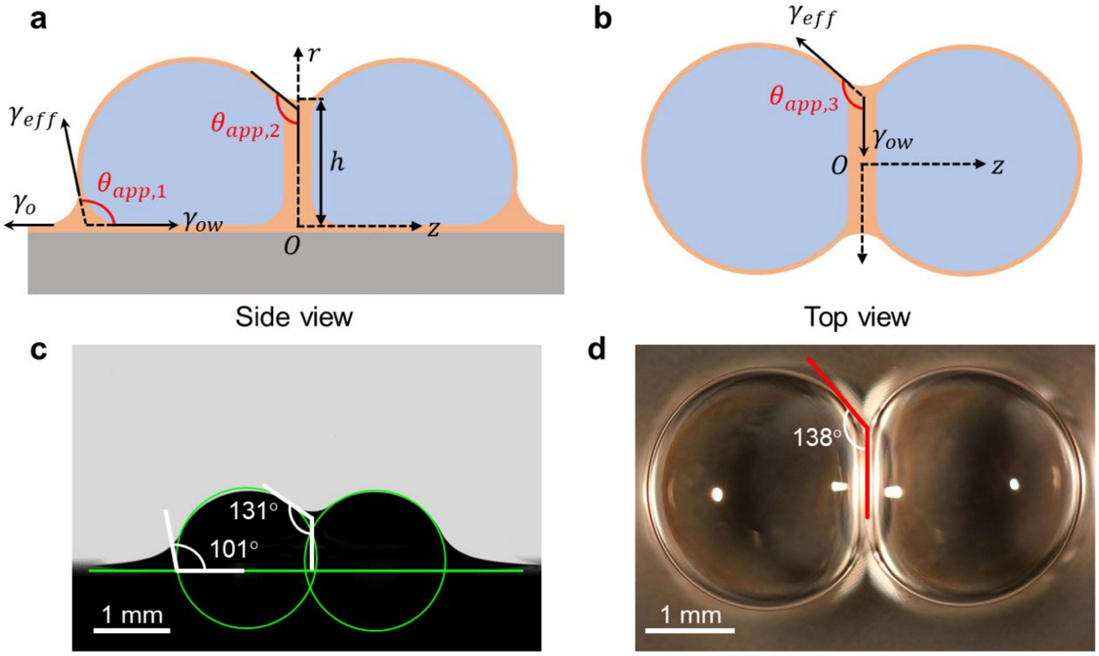

Supplementary Figure 8. **Apparent contact angle. a,b** Schematic side view and top view of two coalescing droplets. **c,d** Actual side view and top view images of coalescing droplets.

When the two droplets coalesce, they first drain the oil from the wetting ridge. This drainage process is captured in our high-speed visualization experiments where an immiscible oil blob was observed to roll down the droplet as shown by the red arrows in Supplementary Fig. 9. The blob of oil sliding downhill from the droplet top is clearly visible in the snapshot taken at 44 ms (see inset, scale bar = 250 μm).

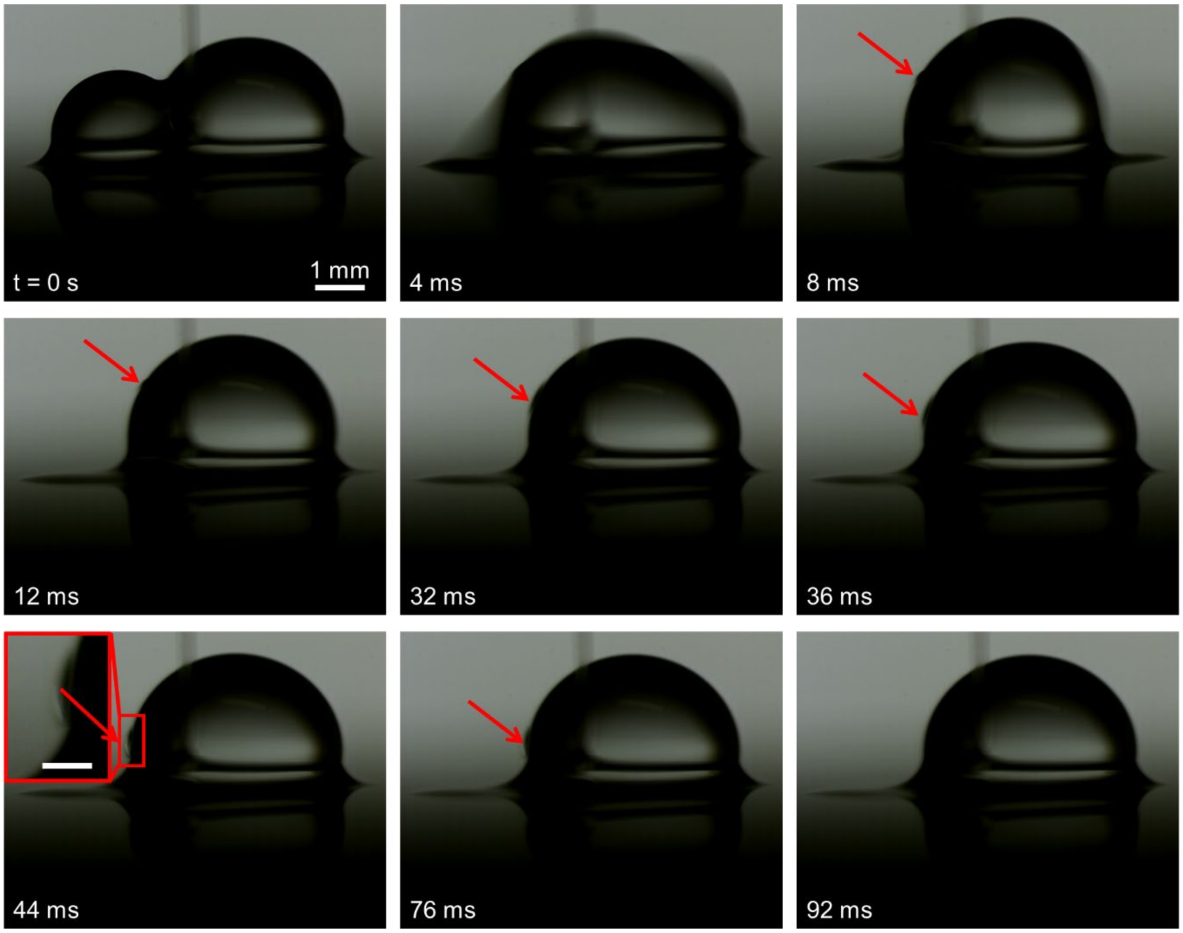

Supplementary Figure 9. **Coalescence mechanism.** Coalescing droplets drain the oil in the wetting ridge before fully merging into one large droplet. The blob of oil that is pushed out of the wetting ridge between the two droplets rolls down the droplet into the wetting ridge of the coalesced new droplet. The scale bar in the inset at t = 44 ms is 250 μm.

S7. Coalescence-induced droplet harmonic oscillation

Until the first peak, the droplets are nearly spherical and the center of mass of the spheres was used to estimate u_1 and a_1. The droplets, however, cannot be approximated as hemispheres after the first peak due to substantial deformation (deviation from a hemisphere approximation). Therefore, we used the change in the cross-sectional area of droplets A (shaded area in Fig. 4a) to model the second velocity and acceleration peaks.

The release of free surface energy during coalescence causes out-of-plane droplet jumping on traditional air-filled superhydrophobic surfaces. Due to the presence of a wetting ridge, however, coalescing droplets on oil-impregnated surfaces do not jump from the surface. Instead, they oscillate back and forth in the horizontal in-plane direction. We modeled this oscillatory motion using the standard mass-spring harmonic oscillator wherein the surface tension and oil viscosity play the roles of restoring force (spring constant) and damper (damping coefficient), respectively.

Analysis of time-lapse images shows that the droplets undergo substantial shape change, that is, both the cross-sectional area (A) and its time derivative ($-dA/dt$) oscillate before they stabilize to an equilibrium position. Area A represents the total/combined cross-sectional area of both droplets along with the respective wetting ridges. Analogous to a standard mass-spring system, the period of oscillation (τ_2) remains approximately the same while the amplitude of oscillation decreases when oil viscosity increases

from 10 cP to 20 cP. Importantly, unlike the first peak, the second peak velocity is insensitive to oil viscosity.

Drawing analogy with the standard damped mass-spring system, the equation governing droplet oscillation during coalescence is given by

$$ma + \eta_o \frac{u}{h_1}(2\pi R d) - \Delta p\left(\pi R \frac{\ell}{2}\right) = 0,$$
$$\ddot{\ell} + \eta_o \frac{2\pi R d}{m h_1}\dot{\ell} + \frac{\Delta p \pi R}{2m}\ell = 0, \tag{8}$$

where m is the mass of the droplet, Δp is the curvature-induced pressure difference on the $z = 0$ plane between the oil in the wetting ridge and the surrounding ambient air ($\Delta p \sim \gamma_{eff}/R$), R is the droplet radius, h_1 is the lubrication film thickness, d is the size of the rim around the droplet base (Fig. 4b), η_o is the oil viscosity, ℓ is half the separation between the droplets, $a = -\ddot{\ell}$, and $u = -\dot{\ell}$. The minus sign in the pressure term $\Delta p(\pi R\ell/2)$ is due to the high pressure that builds up in the oil in the wetting ridge that resists droplet coalescence. The boundary conditions for Supplementary Equation (8) are $\ell(t = 0) = \ell_0$, $\ell(t \to \infty) = 0$, and $\dot{\ell}(t = 0) = 0$, where ℓ_0 is half of the initial separation between the droplets. The solution to Supplementary Equation (8) is given by $\ell = \ell_0 e^{-t/t_{\text{decay}}}\cos(\omega t)$, where t_{decay} is a characteristic decay time. The cross-sectional area $A \approx A_\infty + 2\ell h$, where h is the height of the droplet and A_∞ is the cross-section area after coalescence ($t \to \infty$). Substituting ℓ in A gives $A \approx A_\infty + 2h\ell_0 e^{-t/t_{\text{decay}}}\cos(\omega t)$, which agrees qualitatively with experiments (Fig. 4d). The angular frequency of oscillation is given by $\omega = \sqrt{\frac{\pi \Delta p R}{2m} - \left(\frac{\eta_o \pi R d}{m h_1}\right)^2}$, which simplifies to $\omega = \sqrt{\frac{\pi \Delta p R}{2m}}$ since $\frac{\pi \Delta p R}{2m} \gg \left(\frac{\eta_o \pi R d}{m h_1}\right)^2$. Further simplification of the frequency by using the scaling $m \sim \rho R^3$ and $\Delta p \sim \gamma_{eff}/R$ gives the frequency of oscillation $\omega \sim \left(\rho R^3/\gamma_{eff}\right)^{-1/2}$, which can be rearranged to give the period $\tau_2 \sim \left(\rho R^3/\gamma_{eff}\right)^{1/2}$. This result is validated with experiments in Fig. 4h. Additionally, our experiments show that the decay time t_{decay} for the droplet oscillation scales inversely with oil viscosity ($t_{\text{decay}} \propto \eta_o^{-1}$) (Fig. 4i), a result that agrees with theory ($t_{\text{decay}} \sim \left(\frac{m h_1}{\pi R d}\right)\frac{1}{\eta_o}$).

S8. Measuring second velocity peak by tracking wetting ridge-droplet intersection point

In addition to the area analysis discussed in the main manuscript, we estimated the second velocity peak (u_2) by tracking the intersection point between the droplet and the wetting ridge (arrow, Supplementary Fig. 10a) using MATLAB. The horizontal position (x, Supplementary Fig. 10b) and velocity ($u_2 = dx/dt$, Supplementary Fig. 10c), which behave like an underdamped mass-spring system, show that $u_2 \approx 14$ cm/s. This velocity is higher than the velocity estimated using area analysis. We believe that this method overestimates the velocity since the wetting ridge-droplet intersection point travels an additional distance as the droplet stretches vertically during coalescence. Note that vertical stretching of the droplet causes fluid motion (and by extension, wetting ridge-droplet contact point) in the horizontal direction which results in overestimation of the second peak velocity.

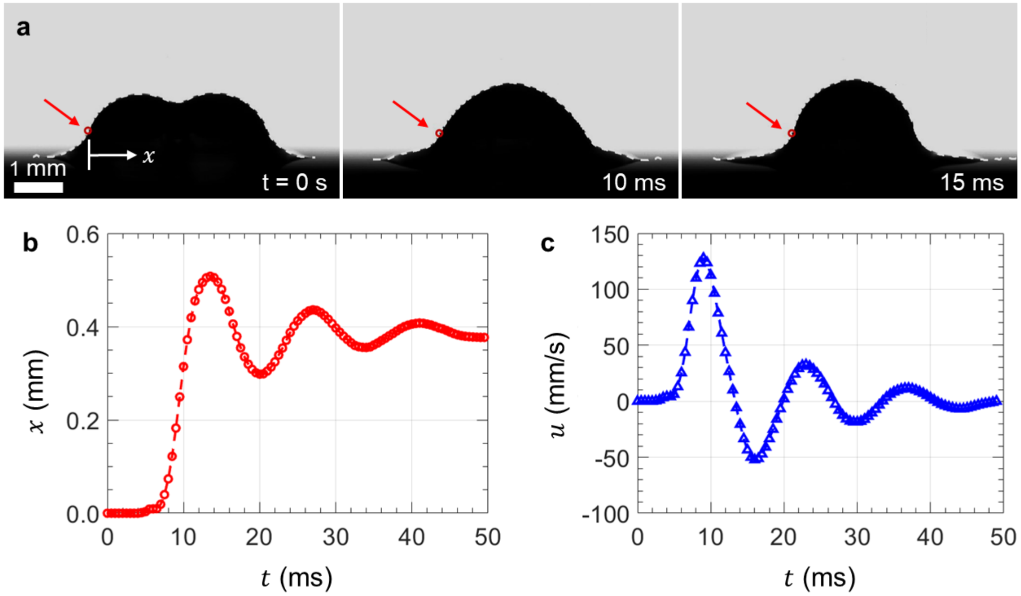

Supplementary Figure 10. **Second peak velocity using edge detection. a** The intersection point between the droplet and the wetting ridge (arrow) is tracked using MATLAB. **b-c** Instantaneous droplet position (x) (**b**) and velocity ($u = dx/dt$) (**c**) as a function of time. Since the wetting ridge-droplet intersection point continues to move horizontally after the center of mass of the droplet has stopped moving in the horizontal direction, this method overestimates the second velocity peak.
