# Supplementary material for: Droplet attraction and coalescence mechanism on textured oil-impregnated surfaces
Source: Nat Commun. 2023 Aug 18;14:4901. doi: 10.1038/s41467-023-40279-w (PMC10439220; doi:10.1038/s41467-023-40279-w)
Supplement: Supplementary file 2 — Description of Additional Supplementary Files [file 41467_2023_40279_MOESM2_ESM.pdf]

Description of Additional Supplementary Files

Supplementary Movie 1. **Droplet attraction (stage I).** Droplet-droplet interaction captured at 10000 fps. In this experiment, both droplets were 5 μl, oil viscosity was 5 cP, and the oil film thickness was 23 μm. The movie is played by slowing the coalescence process by 400×.

Supplementary Movie 2. **Droplet coalescence (stage III).** Droplet coalescence captured at 10000 fps. In this experiment, both droplets were 5 μl, oil viscosity was 5 cP, and the oil film thickness was 23 μm. The movie is played by slowing the coalescence process by 400×.

Supplementary Movie 3. **Second velocity peak measurement by tracking wetting ridge-droplet intersection point.** Droplet coalescence captured at 2000 fps. In this experiment, both droplets were 5 μl, oil viscosity was 10 cP, and the oil film thickness was 23 μm. The movie is played by slowing the coalescence process by 400×.
